# Signatures of Selection in Composite Vrindavani Cattle of India

**DOI:** 10.3389/fgene.2020.589496

**Published:** 2020-12-18

**Authors:** Akansha Singh, Arnav Mehrotra, Cedric Gondro, Andrea Renata da Silva Romero, Ashwni Kumar Pandey, A. Karthikeyan, Aamir Bashir, B. P. Mishra, Triveni Dutt, Amit Kumar

**Affiliations:** ^1^Animal Genetics Division, Indian Council of Agricultural Research (ICAR)-Indian Veterinary Research Institute, Bareilly, India; ^2^Department of Animal Science, Michigan State University, East Lansing, MI, United States; ^3^Department of Animal Science, São Paulo State University, São Paulo, Brazil; ^4^Animal Biotechnology, Indian Council of Agricultural Research (ICAR)-Indian Veterinary Research Institute, Bareilly, India; ^5^Livestock Production and Management, Indian Council of Agricultural Research (ICAR)-Indian Veterinary Research Institute, Bareilly, India

**Keywords:** crossbred cattle, *F*_*ST*_, integrated haplotype score, selection signature, XP-EHH

## Abstract

Vrindavani is an Indian composite cattle breed developed by crossbreeding taurine dairy breeds with native indicine cattle. The constituent breeds were selected for higher milk production and adaptation to the tropical climate. However, the selection response for production and adaptation traits in the Vrindavani genome is not explored. In this study, we provide the first overview of the selection signatures in the Vrindavani genome. A total of 96 Vrindavani cattle were genotyped using the BovineSNP50 BeadChip and the SNP genotype data of its constituent breeds were collected from a public database. Within-breed selection signatures in Vrindavani were investigated using the integrated haplotype score (iHS). The Vrindavani breed was also compared to each of its parental breeds to discover between-population signatures of selection using two approaches, cross-population extended haplotype homozygosity (XP-EHH) and fixation index (*F*_ST_). We identified 11 common regions detected by more than one method harboring genes such as *LRP1B, TNNI3K, APOB, CACNA2D1, FAM110B, and SPATA17* associated with production and adaptation. Overall, our results suggested stronger selective pressure on regions responsible for adaptation compared to milk yield.

## Introduction

The benefits of crossbreeding between high yielding *Bos taurus* and environmentally resistant *Bos indicus* breeds in tropical production systems have been well-established over the last half-century. Crossbred cattle have played an important role in meeting India's rising demand for milk. Despite constituting only 20.7% of India's milch herd, the crossbreds contribute 26% of India's annual milk production of 187.75 metric tons (DAHDF, 2018-19; 20^*th*^ Livestock Census, 2019).

A four breed crossing scheme was initiated at the Indian Veterinary Research Institute in 1968. Briefly, a foundation stock of 400 indigenous Hariana cattle was inseminated with Holstein Friesian (HF), Jersey and Brown Swiss (BSW) semen to produce three genetic groups *viz*., 1/2 Hariana × 1/2 HF, 1/4 Hariana ×1/2 HF ×1/4 BSW, and 1/4 Hariana ×1/2 HF ×1/4 Jersey. These genetic groups were evaluated for production, reproduction and environmental adaptation for seven generations. This was followed by inter-mating and selection to create the present day composite breed Vrindavani, having 25–50% *Bos indicus* and 50–75% *Bos taurus* inheritance (Singh et al., 2011). From the records of the distribution of frozen semen straws of the superior Vrindavani bulls, and the cows auctioned to the farmer, it is estimated that presently about 50,000 Vrindavani cattle are in the field.

Over the last decade, SNP microarrays and whole genome sequencing technology has enabled researchers to explore the genetic architecture and signatures of post-admixture selection in composite breeds (Decker et al., 2014; Kim and Rothschild, 2014; Cheruiyot et al., 2018). In Vrindavani cattle, the Bovine SNP50K array has recently been used to investigate the population structure of the breed (Chhotaray et al., 2019; Ahmad et al., 2020). Since the inception of the Vrindavani breeding program almost five decades ago, the breed has been under selection for milk production and adaptation to tropical conditions. We hypothesized that natural and artificial selection has left footprints on the genome of Vrindavani cattle over the years. Identification of the regions under selection could improve our understanding of the molecular mechanisms driving the environmental adaptation and increased milk production of composite Bos taurus × indicus breeds in the tropics. Therefore, the objective of this study was to detect signatures of selection in the genome of the Vrindavani cattle using two complementary approaches. First, the integrated haplotype score (iHS) was used to detect within-population selection signatures. Second, we compared Vrindavani to Hariana, HF, Jersey and BSW by haplotype based (XP-EHH) and single SNP based (*F*_ST_) methods to discover the genomic regions where the composite breed has diverged from each of its parental populations since the admixture.

## Materials and Methods

### Sample Collection, Genotyping and Quality Control

Blood samples from 96 lactating Vrindavani cattle in lactations ranging from 1 to 6 were collected from Cattle and Buffalo breeding farm of the ICAR-Indian Veterinary Research Institute, Bareilly, UP (28.3670° N, 79.4304° E), following approval by the Institutional Animal Ethics Committee (IAEC). The cows under study were offspring of 16 sires and were born between year 2013 to 2018, with average lactation stage of 176 days. The Vrindavani bulls on the farm were selected and culled on the basis of dam and daughter's milk yield, respectively. Involuntary culling was practiced for cows with mastitis.

Genomic DNA was isolated using Qiagen DNeasy Blood Mini Kit (Qiagen, Valencia, CA) according to the manufacturer's instructions. The quality and quantity of DNA were evaluated using NanoDrop spectrophotometer, agarose gel electrophoresis and Qubit fluorometer. The extracted DNA samples were genotyped with the BovineSNP50 v3 BeadChip (Illumina, Inc.) using manufacturer's protocols (AgriGenome Labs Pvt. Ltd., India) consisting of 53,218 SNPs across the genome at a mean distance of 37.4 kb. Genotypes were called and processed using the GenomeStudio software package (Illumina, Inc.). The SNP coordinates followed the ARS-UCD1.2 assembly of the bovine genome.

Data of all the 96 Vrindavani animals were used for population structure analysis and within-breed signatures of selection (iHS). Quality filtering of data was performed using PLINK v1.9 (Purcell et al., 2007) by filtering non-autosomal and unmapped SNPs. SNPs with less than a 90% call rate, minor allele frequency lower than 0.01 and a significant (*P* < 0.00001) deviation from Hardy-Weinberg equilibrium were also removed, leaving a dataset of 41,342 SNPs. After filtering, the total genotyping rate was 99.81%, and no individual was removed for missing genotypes.

### Population Structure Analysis

The expected heterozygosity (He), observed heterozygosity (Ho) and minor allele frequency (MAF) was estimated using PLINK 1.07 (Purcell et al., 2007). Principal Component (PCA) and Admixture (Alexander et al., 2009) analyses were performed to validate the breed separation in our merged dataset. The results were visualized in R with the basic plot function (R Core Team., 2018).

### Selection Signature Analyses

The within population signatures of selection in Vrindavani (*n* = 96) were computed using the integrated haplotype score (iHS) (Voight et al., 2006). The ancestral allele information for the iHS test was obtained from Rocha et al. (2014) for the 50K SNP data. The iHS was calculated for each autosomal SNP in Vrindavani through the package rehh (Gautier et al., 2017). Candidate regions were identified using a scan window of 100 kb with a 50 kb overlap. Windows with an average iHS score of 3 (three standard deviations above the mean) or above were considered as candidate regions for selection.

To ascertain between-population selection signatures, Vrindavani was compared to each of its parental populations using XP-EHH (Sabeti et al., 2007) and *F*_ST_ (Weir and Cockerham, 1984).

The genotypic data of Vrindavani's parent taurine breeds (BSW, HF, Jersey) were accessed using WIDDE (http://widde.toulouse.inra.fr/widde/widde/main.do?module=cattle) and Hariana cattle through KRISHI (https://krishi.icar.gov.in/jspui/handle/123456789/31167) web portals. These included 50K SNP data of HF (*n* = 30), Jersey (*n* = 21), BSW (*n* = 24), and the HD (777K) genotypes of Hariana (*n* = 18). The common SNPs between 50K and HD chip data of Hariana were extracted for further analysis. Since large differences between the sample sizes of the groups under comparison can cause inaccurate *F*_*S*__T_ estimates (Barendse et al., 2009; Bhatia et al., 2013), a subset of 25 Vrindavani animals was used for the across-population comparisons. We calculated the pairwise identity by state (IBS) scores for all the 96 Vrindavani animals using PLINK, and retained the 25 animals with the least amount of shared similarity (IBS).

Genotypic data of all the breeds were merged and quality control was performed again by using the settings mentioned above leaving 34,197 variants for downstream analysis. The genotypes were phased using BEAGLE v5.1 (Browning et al., 2018) using default settings (burnin = 6; iterations = 12; phase-states = 280).

The XP-EHH scores were calculated for each pairwise comparison using the package rehh, taking the parental breeds as the reference population. To detect positive selection in Vrindavani, average XP-EHH scores were computed for 100-kb regions with a 50 kb overlap. Regions with absolute XP-EHH scores of 3 (Three SD above the mean) or above were considered as putative candidate regions. The pairwise *F*_*ST*_ was calculated with VCFTOOLS (Danecek et al., 2011), with a sliding window of 100 kb and a 50 kb step size. Windows belonging to the top 0.1% of the *F*_ST_ values were considered as potential regions under selection. The genes present in the candidate regions were annotated using the Ensembl Biomart genes database (release 100). Functional and pathway enrichment analysis was performed using DAVID (Huang et al., 2009). Each positively selected region was cross referenced with the literature.

## Results

### Descriptive Statistics and Population Structure Analyses

**Figure 1:**
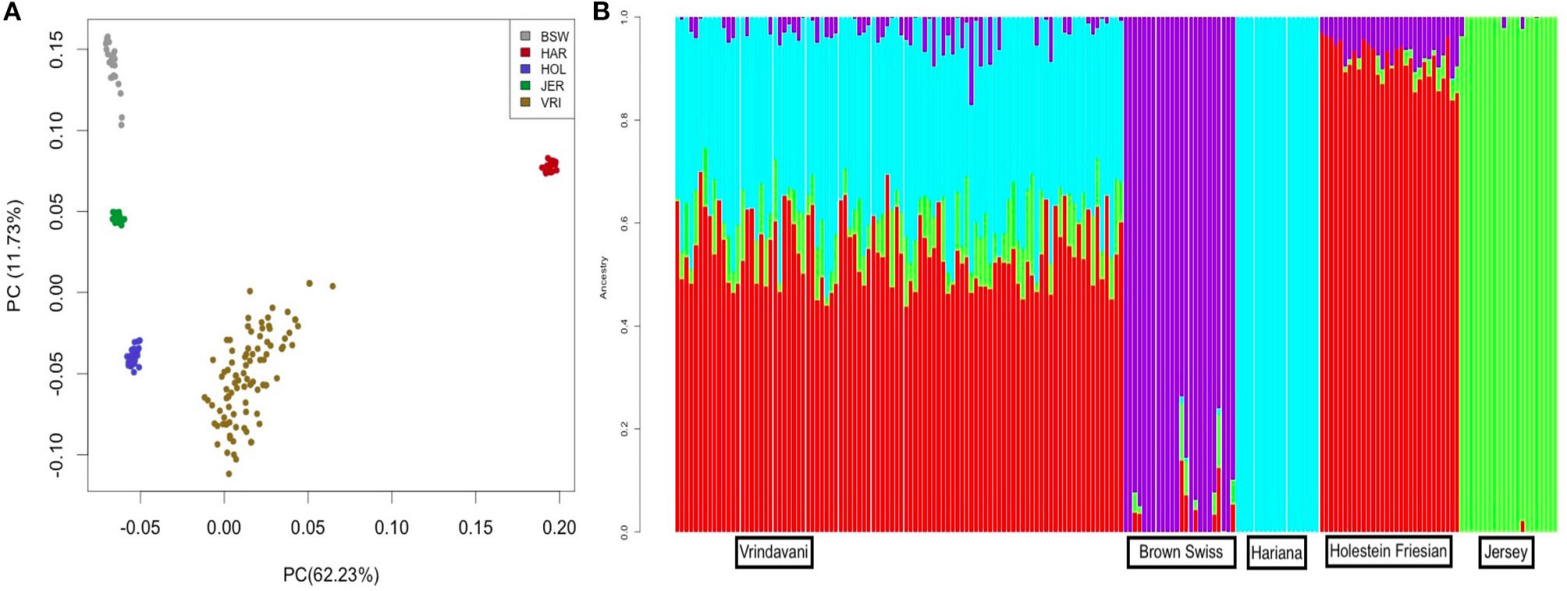
**(A)** Principal Component Analysis (PCA) plot showing clustering of Vrindavani crossbred cattle and parent breeds. (BSW, Brown Swiss; HAR, Hariana; HOL, Holestein; JER, Jersey; VRI, Vrindavani). **(B)** Admixture analysis of Vrindavani cattle showing proportion of ancestral population (K = 4).

The heterozygosity and MAF values of Vrindavani (*Ho* = 0.34, MAF = 0.28) were found similar to the European breeds, particularly to HF (Table 1). The genetic relationship between the Vrindavani population and its parent breeds was visualized using PCA. The first and second principal components explained 62.3% and 11.7% of the total variation, placing the Vrindavani cattle in between the taurine and indicine breeds which is in agreement with their known lineage (Figure 1A). They are however noticeably closer to the Holstein cluster than any of the other parental breeds. In concordance with Ahmad et al. (2020), Admixture analysis with *K* = 4 showed that the average breed composition proportions for our population of Vrindavani was 42.5%, 26.0%, 17.1%, and 14.4% of HF, Hariana, Jersey and BSW, respectively (Figure 1B).

**Table 1:** Number of animals, means of observed (H_O_) and expected heterozygosity (H_E_), minor allele frequencies (MAF) and differentiation (*F*_ST_) between each breed with Vrindavani.

**Breed**	***N***	**H_**O**_ (Mean ± SD)**	**H_**E**_ (Mean ± SD)**	**MAF**	**F_**ST**_**
Vrindavani	96	0.38 ± 0.13	0.34 ± 0.11	0.28	–
Brown Swiss	24	0.31 ± 0.20	0.29 ± 0.17	0.22	0.13
Jersey	21	0.31 ± 0.19	0.26 ± 0.17	0.23	0.14
Holestein-Friesian	30	0.35 ± 0.16	0.34 ± 0.15	0.26	0.08
Hariana	18	0.29 ± 0.18	0.28 ± 0.15	0.20	0.24

### Within Population Selection Signatures in Vrindavani (iHS)

**Figure 2:**
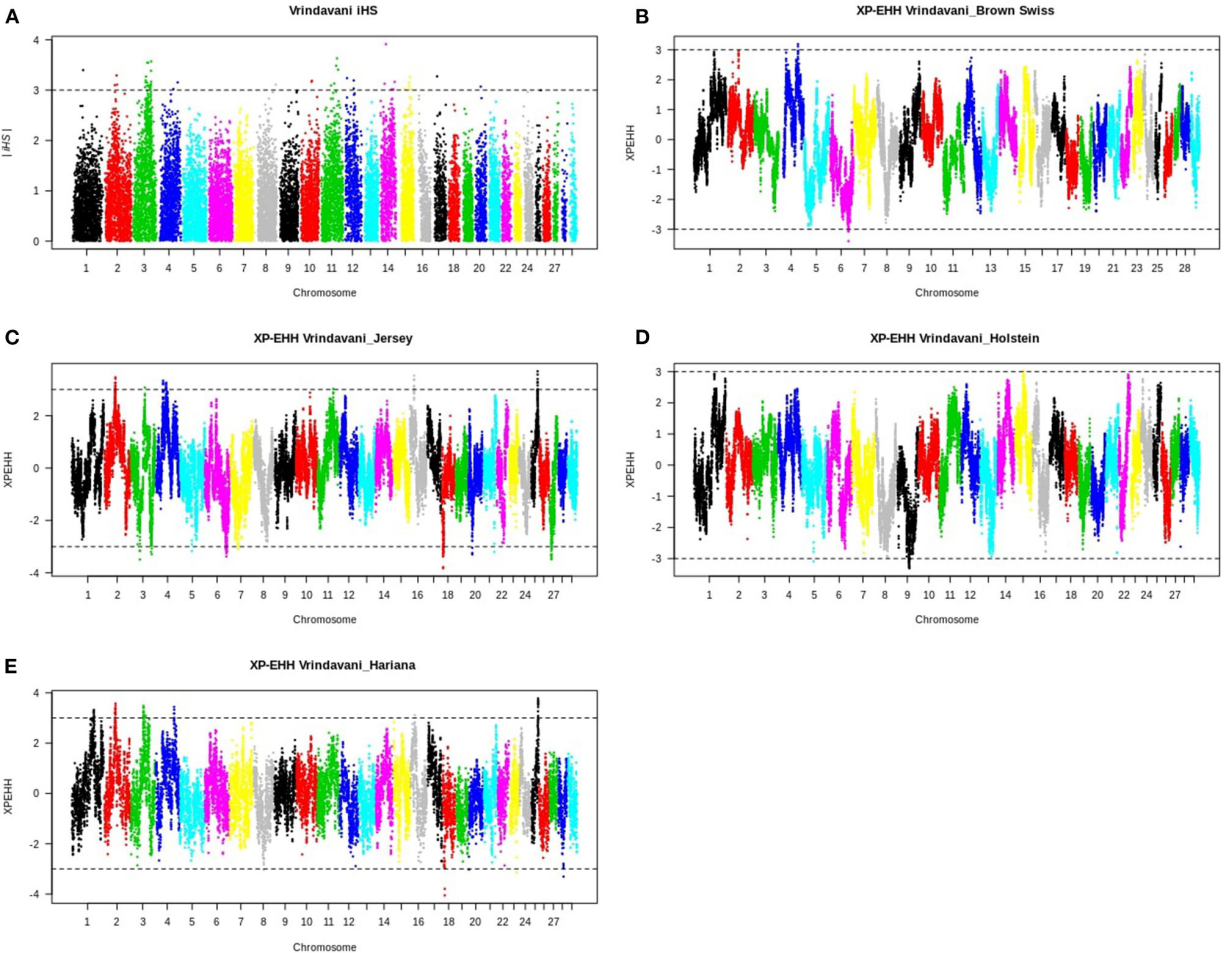
**(A)** Plot of the integrated haplotype score (iHS) plot for the *Vrindavani* cattle. **(B–E)** Cross-population extended haplotype homozygosity (XP-EHH) plots for Vrindavani's comparisons with **(B)** Brown Swiss, **(C)** Jersey **(D)** Holstein, and **(E)** Hariana. The dotted lines indicate mean ± 3 standard deviations as threshold.

Considering the recent selection history in Vrindavani breed, the selection sweeps were identified using integrated haplotype score (iHS) approach. A total of 46 significant SNPs (iHS ≥3) distributed across 12 autosomes were identified within the candidate regions (Figure 2A, Supplementary Table 1). The strongest iHS signal (3.9) was found on BTA14 (30.35–30.44 Mb). The top 10 regions with their iHS values and genes are shown in Table 2. Functional annotation of the selected regions identified candidate genes related with milk production (*APOB, ANO3, DNMT3A*, and *POMC*) and environmental adaptations or immunity (*DNAJC5B* and *FYB2*).

**Table 2:** List of the top 10 regions identified by the integrated haplotype score measures (iHS), and the genes present within them.

**BTA**	**Start**	**End**	**iHS**	**Gene**
14	30,355,171	30,448,182	3.913994	DNAJC5B
11	77,798,022	77,927,967	3.631122	TDRD15, APOB
3	89,219,877	89,464,197	3.570568	C8A, FYB2
3	75,039,363	75,644,478	3.545228	LRRC7
11	73,971,560	74,224,519	3.483963	DNMT3A, POMC, EFR3B
11	82,677,576	83,207,199	3.404882	DDX1, NBAS
1	57,570,680	57,681,112	3.398117	CD200R1L
3	88,677,121	89,136,086	3.383668	DAB1
2	61,535,026	61,675,889	3.291714	LCT, UBXN4, R3HDM1, MIR128-1
3	65,667,175	65,807,163	3.270202	ADGRL4

### Across Population Selection Signatures (*F_*ST*_* and XP-EHH)

The Manhattan plots of pairwise XP-EHH analysis between Vrindavani and its parent breeds are presented in Figures 2B–E, and the information about the significant regions is in Supplementary Table 2. The selection signals (positive value of XP-EHH for Vrindavani), against all parent breeds were detected on 8 autosomes, of which clusters of SNPs are observed on BTA1, BTA2, BTA3, BTA4, BTA11, BTA15, BTA16, and BTA25. BTA4 and BTA3, exhibit the highest number of selected regions in all the breed comparisons. The selective sweep located on BTA4 (91.8–92.2 Mb) was detected in comparisons with both BSW and Hariana. It contains the genes *SND1* and *LRRC4* associated with somatic cell count, milk yield and residual feed intake. Two regions on BTA2 (56.4–56.6 Mb) and BTA25 (31.15–31.3 Mb) were detected in comparisons with Hariana and Jersey. It contains *LRP1B* gene and QTLs for somatic cell count and reproduction traits (Cole et al., 2011).

The mean *F*_ST_ values of Vrindavani in comparison to HF, Jersey, BSW and Hariana were 0.081, 0.110, 0.122, and 0.175, respectively. The pairwise F_ST_ across genome of the Vrindavani against its parental breeds were plotted in Supplementary Figure 1. A total of 124 regions were identified, which were distributed across all autosomes except BTA25 and BTA26 in breed comparisons (Supplementary Table 3). The regions on the chromosomes having highest *F*_ST_ values against HF, BSW, Jersey and Hariana were located on BTA4, BTA7, BTA10, and BTA3, respectively.

The *F*_ST_ signals on overlapping regions located on BTA7 (45.3–48.9 Mb), BTA10 (37.4–37.7 Mb) and on BTA14 (12.3–12.6 Mb) were observed in comparison with BSW and Jersey, harboring genes involved with production, reproduction and functional traits (*H2AFY, SPOCK1, PLA2G4D, PLA2G4F*, and *GANC*). Two selected regions on BTA14 from 12.3 to 12.6 Mb (against BSW and HF) and 27.3–27.9 Mb (against Jersey and HF) were identified. These regions include *SPIDR* gene (Scaffold protein involved in DNA repair) associated with milk and milk protein yield) and *NKAIN3* gene (Na^+^/K^+^ transporting ATPase interacting 3) related with insulin-like growth factor one level.

### Comparative Analysis of Selection Signatures

A total of 13 regions on BTA2, BTA3, BTA4, BTA10, BTA11, BTA14, BTA15, and BTA16 were determined by more than one approach; with a region on BTA3 (70.2–72.2 Mb) common to all three approaches (Table 3). Out of six regions detected by both the between population approaches (pairwise *F*_ST_ and XP-EHH), four regions were detected in comparisons with taurine breeds (BSW, Jersey and HF); one region in comparison with the indicine breed (Hariana) and one region against both taurine and indicine breeds (Table 3). Functional annotation of the commonly detected regions shows several candidate genes already reported as selection signals or associated with economic traits in different cattle breeds. Genes present in these regions enriched biological processes such as response to virus (GO:0009615) and post embryonic development (GO:0009791), and molecular functions such as ATP binding (GO:0005524) and motor binding (GO:0003774) shown in Supplementary Table 4.

**Table 3:** Selection sweeps identified by more than one test in the Vrindavani chromosomes (BTA) and annotated genes in these regions.

**Test**	**BTA (Start-End Mb)**	**Annotated genes**
XPEHH(Hariana), *F*_ST_(Hariana); iHS	3 (70.2–72.18)	TNNI3K
XPEHH(Jersey, Hariana); *F*_ST_(HF)	2 (55.5–56.7)	LRP1B
XPEHH(Jersey); *F*_ST_(HF)	4 (38.1–40.1)	CACNA2D1, 7SK,SEMA3C
XPEHH(Jersey); *F*_ST_(HF)	4 (52.28–53.65	TFEC
XPEHH(HF); *F*_ST_(Jersey)	15 (43.3–44.1)	TRIM66, STK33, DENND5A, SCUBE2, NRIP3
XPEHH(Jersey); *F*_ST_(BSW)	16 (21.1–22.9)	SPATA17, RRP15
XPEHH(Jersey); iHS	11 (77.3–78.0	TDRD15, APOB
XPEHH(Hariana); iHS	3 (64.1–65.8)	ADGRL4/ELTD1
*F*_ST_(BSW); iHS	10 (5767–58.20)	MYO5A,bta-mir-1248-2, MYO5C
*F*_ST_(HF); iHS	14 (24.36–24.62)	FAM110B,UBXN2B
*F*_ST_(Hariana); iHS	11 (82.67–83.20)	DDX1, NBAS

## Discussion

The Admixture analysis and PCA plot reflected the presence of both indicine and taurine ancestry in our Vrindavani population, with a higher proportion of taurine ancestry (Holstein). The dominance of the Holstein component in Vrindavani cattle has also been recently reported in a different set of Vrindavani population (Ahmad et al., 2020).

In the present study, we wished to evaluate the effect of natural and artificial selection on the Vrindavani genome, compared to its parent breeds. Due to genetic drift (Akey et al., 2002), and ascertainment bias of the SNP chip toward taurine breeds, it is difficult to distinguish true signatures of selection from false positives in crossbred cattle. Thus, three different methods of signature of selection (iHS, *F*_ST_, and XP-EHH) were applied with stringent thresholds to capture putative regions of selection across the genome.

The regions commonly detected by pairwise cross population methods (XP-EHH and *F*_ST_) against taurine breeds on BTA4 contained the *CACNA2D1* gene, which is a member of the calcium voltage-gated channel auxiliary subunit alpha-2/delta. It is previously reported to be a candidate gene associated with somatic cell score (Deng et al., 2011) and mastitis resistance (Yuan et al., 2011). Another gene on this chromosome is *TFEC*, reported to be a selection signature in African cattle and related with resistance to ticks and other tropical disease (Tijjani et al., 2019).

On BTA14, *FAM110B*, and *UBXN2B* genes were identified to be associated with productive traits, reproductive traits (Grigoletto et al., 2019) and feed efficiency (Seabury et al., 2017). Flori et al. (2009) has also reported *FAM110* as selection signature for dairy cattle under artificial selection. The *STK33* gene on BTA15 reported in the *F*_ST_ and XP-EHH analyses against the Jersey and HF cattle, respectively, were reported as selection signatures in Gir cattle and are associated with milk production in indicine cattle (Maiorano et al., 2018).

Commonly identified regions from iHS and cross-population approaches against Hariana contain *TNNI3K* and *ADGRL4*/*ELTD1* genes on BTA 3. *TNNI3K* is a cardiac troponin interacting kinase, associated with udder depth (Kramer et al., 2014), inflammation mechanisms (Wiltshire et al., 2011) and lameness in Holstein–Friesian cattle (Sánchez-Molano et al., 2019). The *ADGRL4/ELTD1* gene is associated with milk fat yield (Li et al., 2010) and tick resistance (Porto Neto et al., 2011) in dairy cattle. Another gene *DDX1* on BTA11 was reported to be involved in bovine mammary involution in environmental stress conditions (Dado-Senn et al., 2018). *DDX1* is also reported to be associated with linoleic acid content in Nellore cattle (Lemos et al., 2016) and viral resistance (Xue et al., 2019). A common region in comparisons with both taurine and indicine breeds is detected on BTA2. It harbors *LRP1B* gene which codes for low density lipoprotein related with milk yield (Chen et al., 2015) and somatic cell score (Cole et al., 2011). *LRP* is widely expressed in several tissues and plays important roles in lipoprotein catabolism, blood coagulation, cell adhesion and migration (Haas et al., 2011).

Overall, the results revealed that selection was operative more strongly in the regions related to environmental adaptation than milk yield, despite the latter being a focus of artificial selection. This could be explained by the presence of a large (50–75%) taurine inheritance in the Vrindavani genome, so a deviation from the parental breeds with respect to adaptation was not unexpected.

The slow rate of genetic gain with respect to the artificially selected productivity traits, due to the small and closed nature of the institutional herd examined in this study may also be responsible for our findings. Vrindavani is still a relatively new breed, and we expect these selection signatures to be more prominent in the coming generations.

## Conclusion

This study provided the first overview of the selection footprints in the genome of the composite Vrindavani cattle of India. The signatures of selection for Vrindavani breed reveals several genomic regions which were involved in milk production and adaptation. Our results confirmed some of the key candidate genes such as *CACNA2D1, DDX1*, and *ADGRL4* which were known to be previously associated with immune related and adaptation pathways. Interestingly, the findings suggest that selection in Vrindavani was operative more strongly in the regions related to environmental adaptation than milk yield, despite the latter being a focus of artificial selection. To further reduce false positives and increase the resolution of detection of selection signatures, we suggest validation of this study in a larger field herd using the HD genotyping array or whole-genome sequence data.

## Data Availability Statement

The datasets presented in this study can be found in online repositories. The names of the repository/repositories and accession number(s) can be found below: https://figshare.com/, https://doi.org/10.6084/m9.figshare.12808343.v2.

## Ethics Statement

The animal study was reviewed and approved by Institute Animal Ethics Committee, Indian Veterinary Research Institute, India.

## Author Contributions

AKu: conceived and designed the experiments. AS and AP: performed the experiments. AM, AB, and AKa: analyzed the data. CG, AR, and TD: contributed reagents/materials/analysis tools. AS, AM, and AKu: wrote the paper. AKu and BM: edited the paper. All authors contributed to the article and approved the submitted version.

## Conflict of Interest

The authors declare that the research was conducted in the absence of any commercial or financial relationships that could be construed as a potential conflict of interest.
