# Supplementary material for: Signatures of Selection in Composite Vrindavani Cattle of India
Source: Front Genet. 2020 Dec 18;11:589496. doi: 10.3389/fgene.2020.589496 (PMC7775581; doi:10.3389/fgene.2020.589496)
Supplement: Supplementary file 2 [file Data_Sheet_2.docx]

**Signatures of Selection in Composite *Vrindavani*** **Cattle of India**

Akansha Singh ^1^, Arnav Mehrotra^1^, Cedric Gondro ^2^, Andrea Renata da Silva Romero^3^, Ashwni Kumar Pandey^1^, Karthikeyan A^1^, Aamir Bashir^1^, B P Mishra^1^,

Triveni Dutt^1^ Amit Kumar ^1*^

1. Animal Genetics Division, ICAR-Indian Veterinary Research Institute, Izatnangar, Bairelly , India
2. Department of Animal Science, Michigan State University, Department of Animal Science, East Lansing, Michigan, USA.
3. Department of Animal Science, São Paulo State University, São Paulo, Brazil

*** Correspondence:**Amit Kumar
vetamitchandan07@gmail.com

Supplementary Figures:

Figure S1: Plot of Fixation index (*F*_ST_) for (a)Brown Swiss, (b) Holstein Friesian (c) Jersey (d) Hariana against the Vrindavani.
